# T116. CAFFEINE-INDUCED PSYCHIATRIC MANIFESTATIONS

**DOI:** 10.1093/schbul/sby016.392

**Published:** 2018-04-01

**Authors:** Kwanghun Lee, Won-Myong Bahk, Bo-Hyun Yoon, Duk-In Jon, Sang-Yeol Lee, Moon Doo Kim, Beomwoo Nam, Min-Kyu Song

**Affiliations:** 1 Dongguk University Hospital; 2 Yeouido St. Mary’s Hospital, The Catholic University of Korea; 3 Naju National Hospital; 4 Hallym University; 5 Wonkwang University, School of Medicine; 6 School of Medicine, Jeju National University; 7 School of Medicine, Konkuk University; 8 Yesan Mental Health Clinic

## Abstract

**Background:**

The association between caffeine consumption and various psychiatric manifestations has long been observed.

**Methods:**

We present two cases that show the ability of caffeine to induce psychotic and manic symptoms, and we also review the extant literature on caffeine-induced psychiatric manifestations.

**Results:**

On the basis of our own and others’ findings, we suggest that caffeine may be related to not only de-novo psychotic or mood symptoms but also to aggravation of pre-existing psychotic or mood disorders.

**Discussion:**

We therefore suggest that caffeine consumption among patients with mood or psychotic symptoms should be assessed carefully in clinical practice as part of routine psychiatric evaluations.

